# Preserving the brain: forum on neurodegenerative diseases

**DOI:** 10.1007/s10072-023-06721-z

**Published:** 2023-04-01

**Authors:** Giancarlo Comi, Letizia Leocani, Fabrizio Tagliavini

**Affiliations:** 1 Department of Neurorehabilitation Sciences, Casa Di Cura Igea, Milan, Italy; 2 University Vita-Salute San Raffaele and Experimental Neurophysiology Unit, Institute of Experimental Neurology (INSPE), Scientific Institute San Raffaele, Milan, Italy; 3 Fondazione IRCCS Istituto Neurologico Carlo Besta, Milan, Italy

On 6 and 7 October 2022, more than 70 experts gathered at the Milan venue of Fondazione Prada for the Preserving the Brain Forum, to explore the topic of neurodegenerative diseases (NDDs) from different angles, starting with an up-to-date exchange of knowledge on epidemiological and genetic implications and molecular disease mechanisms, and ending with a debate on key concepts and possible gaps in clinical trials and promising targets for new treatments.

Neurodegeneration is a central aspect of a wide range of diseases, including Alzheimer’s disease (AD), Parkinson’s disease (PD), amyotrophic lateral sclerosis (ALS) and multiple sclerosis (MS), which are widespread and as yet incurable. NDDs pose one of the greatest challenges to human health in the twenty-first century, striking the most complex organ of the human body and causing enormous damage to some of the most fundamental human traits, including memory and personality. The current understanding of these disorders builds upon decades of advances in neuroscience and clinical trials. This highly scientific conference addressed researchers and universities involved in the Human Brains project and representatives of patients’ organizations, healthcare institutions and pharmaceutical and biotechnology industries.

Neurological diseases affect one out of three people worldwide, the main driving forces being NDDs [1]. Epidemiological transition of the ageing population adds to the growing burden of NDDs, particularly when coupled with unhealthy lifestyles (e.g. lack of exercise, smoke, inadequate diet, and poor sleep). As such, the contrast to NDDs is fundamental for brain health.

NDDs are complex diseases due to a variable combination of genetic, epigenetic and environmental factors [2–4], the latter including pollution, lifestyles and infections. The recent evidence on the key pathogenetic role of Epstein Barr virus in MS [5] and of the gut brain axes in many neurodegenerative processes [6] have clear treatment implications. The small proportion of monogenic forms present in most of NDDs have provided a wealth of information on the biochemical, molecular, and neural pathways involved in specific disorders [7]. In sporadic cases, identification of genetic risk variants has been facilitated by genome-wide association studies (GWAS) and meta-analyses. Polygenic risk scores developed from GWAS have been proposed to predict heritability in individual subjects and can be used for prevention strategies [8].

The understanding of the molecular and cellular mechanisms of many NDDs is still patchy, which is essential to identify potential treatment targets and strategies. Recent advances in pathological mechanisms linked to ageing were reviewed at this meeting, including loss of the ability to maintain a functional proteome (or proteostasis), mitochondrial dysfunction, disrupted axonal transport and neuroinflammation [9–12]. For decades, it was widely believed that the brain is secluded from peripheral immune activity and is self-sufficient for its maintenance and repair. More recently, neuroinflammation and systemic immune responses have been shown to actively contribute to disease progression in all NDDs and it is important to clarify the role of innate immune system, more specifically the role of microglia [10]. Rejuvenation of the systemic immune system, such as vaccination with CNS antigens and blocking inhibitory PD-1 immune checkpoints, is being explored in mouse models to combat cognitive impairment in AD, for example [13]. Synapse dysfunction and plasticity loss are crucial to the pathophysiology of MS and AD. In the cascade of AD pathogenesis, synaptic failure, triggered by Aβ and tau, has been identified as a key step and an early event in AD, preceding neurodegeneration. Available data on tackling early stages of the disease be used as a guide to find new therapeutic avenues [14].

The focus during the second day was on translating the knowledge on mechanistic aspects to the clinic. The ideal clinical trial is a well-conducted study with appropriate clinical and biomarker outcomes collected over an appropriate period of time, powered to detect a clinically meaningful drug-placebo difference, and anticipating variability introduced by globalisation. As highlighted in the previous sessions, lack of understanding of some critical aspects of disease biology and drug action may affect the success of development programs even when the “rights” are adhered to [15]. Lessons learned from clinical trials, even the failed ones, were discussed. In the past decade, clinical trials have increasingly focused on earlier, pre-NDD stages, as addressing NDD pathology before its onset may be more efficacious for certain interventions, and slowing the disease while the individual is still highly functional is an important goal [16]. Interventions on the prodromal phase of the disease are ongoing or into consideration in MS, PD, AD and Huntington’s disease [17].

Among NDDs, amyotrophic lateral sclerosis (ALS) is the most rapid to progress to fatality, and the development of new therapeutic agents for ALS has been limited by the complexity and heterogeneity of disease pathophysiology. Adaptive platform trials, as a more efficient alternative to randomized controlled trials that test one investigational product at a time, represent a novel approach and have found their way in ALS drug development, and hopefully, many other NDDs will follow [18].

Significant efforts are being made to identify ideal biomarkers that are sensitive to preclinical/prodromal stages, detect disease progression and predict underlying pathology, but can also accelerate clinical trials. A session of this conference covered the latest advances in the pursuit of highly specific but affordable and easy-to-apply biomarkers. The role of conventional and new developed neuroimaging techniques and body fluid biomarkers was debated, with special attention to blood neurofilaments [16, 19–21].

Although several promising therapies are currently being investigated in clinical trials, there is an urgent need for treatments that significantly alter the course of NDDs; most therapies are currently symptomatic. Since NDDs are estimated to consume a large part of the health budget, it is imperative to identify more effective, disease-modifying therapies. In the last two sessions of the conference, therapeutic strategies for NDDs in the third millennium were actualised. One of the greatest challenges in treating NDDs is partly related to the difficulties in penetrating the blood brain barrier (BBB). In many cases, interrogation of early-stage data has not been adequately pursued. Agents have been advanced to phase 3 with little or no evidence of efficacy in phase 2 [15].

Very recently, amyloid antibody-based immunotherapies in AD show evidence of modification of the disease, albeit modestly and at an early stage [22]. Other promising targets in AD include modulation of peripheral inflammation (PD-L1) or suppression of tau by active immunisation or monoclonal antibodies [23]. Novel promising approaches to slow down the progression of PD include interfering with α-synuclein via antibody immunisation, inhibition of α-synuclein and misfolding [24], and targeting the immune system by glucagon-like peptide-1 (GLP-1) receptor agonists that inhibit activated microglia [25]. The treatment paradigm in MS has shifted since the introduction of B-cell immunotherapeutics, showing that B lymphocytes play a central role in the pathogenic cascade [26]. Nevertheless, progression occurs in MS patients across the disease continuum, probably due to chronic CNS inflammation trapped behind an intact BBB. There are ongoing phase 3 clinical trials with agents targeting microglia, Bruton tyrosine kinase receptors, and remyelination [27]. When available, neuroprotective strategies should be associated to anti-inflammatory treatments in the early stages of the disease [28].

Alternative treatments that were discussed at the meeting include cellular therapies [29, 30], gene therapies [29] and neuromodulation with magnetic and electrical stimulation [31, 32]. All these techniques have provided some interesting results, mostly in small-size uncontrolled clinical trials; further validation in larger well-performed studies is necessary.

## Conclusions

The Forum closed with a list of shared priorities for accelerating research to develop solutions for neurodegenerative diseases, reported in Table 1. The key aspect is the collaboration across research centres and across stakeholders. The value of cross-fertilization of experiences matured in the various neurodegenerative diseases has been emphasized as an important achievement of the Forum, to be continued. The involvement of patients in all phases of research was also considered as a fundamental progress: science from and with patient inputs is of great value today. The presence at the Forum of patients and patients’ organizations was a clear evidence in this direction. The future steps of Preserving the Brain Fondazione Prada will ensure the continuity according to the defined priorities.

**Table 1:** Agenda for future actions (agreed priorities)

Assessment of social burden and costs of no treatment
Shared agenda among stakeholders
Collaboration across scientific sectors and across countries
Cross-fertilization across diseases
Attract new generation of scientists
Infrastructures to facilitate access to research
Engagement of patients (science with and of patients inputs)
Professional medical centres
Effects of ageing
Gene environment interactions (more computational power)
Central role of microglia
Primary prevention (physical exercise, diet, sleep, smoking/pollution)
Focus of prodrome phase
Digital medicine
Development and validation of biomarkers, new clinical trial designs
Rehabilitation, brain plasticity
